# Evaluating Surgical Strategies for Adolescent Idiopathic Scoliosis: A Meta-Analysis of Posterior Spinal Fusion and Vertebral Body Tethering

**DOI:** 10.7759/cureus.90739

**Published:** 2025-08-22

**Authors:** Daniel E Onobun, Rahim Hussain, Gerald Lim, Ethel O Ojo, Chijioke Orji, Althea O George, Ajibola A Adebisi

**Affiliations:** 1 Orthopaedics and Trauma, Warwick Hospital, South Warwickshire University NHS Foundation Trust, Warwick, GBR; 2 General Surgery, Worcestershire Acute Hospitals NHS Trust, Worcester, GBR; 3 Orthopaedics, Liverpool University Hospitals Foundation Trust, Liverpool, GBR; 4 Surgery/Urology, The Royal London Hospital, London, GBR; 5 General Surgery, Surgery Interest Group of Africa, Lagos, NGA

**Keywords:** adolescent idiopathic scoliosis, motion preservation surgery, posterior spinal fusion, spinal deformity correction, surgical outcomes, vertebral body tethering

## Abstract

Adolescent idiopathic scoliosis (AIS) is a three-dimensional spinal deformity commonly affecting adolescents worldwide. Surgical management traditionally involves posterior spinal fusion (PSF), which achieves stable deformity correction but may limit spinal motion and growth potential. Vertebral body tethering (VBT) has emerged as a motion-preserving alternative aimed at modulating spinal growth while correcting curvature.

This systematic review and meta-analysis compared PSF and VBT in terms of deformity correction, complication and revision rates, and postoperative functional outcomes. Data from multiple clinical studies were analyzed using standardized methods for pooling and evaluating outcomes. Results indicated that PSF generally provides more consistent and reliable curve correction with fewer complications and revisions, while VBT offers advantages in preserving motion and facilitating earlier postoperative recovery.

However, VBT demonstrated greater variability in results and a higher likelihood of requiring additional interventions. These findings highlight the need for individualized treatment planning that considers curve severity, skeletal maturity, and long-term functional goals. PSF remains a robust corrective option, whereas VBT represents a promising motion-sparing approach for carefully selected patients. Further research is warranted to refine patient selection criteria and optimize long-term outcomes of non-fusion techniques.

## Introduction and background

Adolescent idiopathic scoliosis (AIS) is the most prevalent type of scoliosis in the pediatric population, affecting adolescents aged between 10 and 18 years globally [1]. Scoliosis is characterized by a lateral curvature of the spine exceeding 10 degrees, representing a three-dimensional spinal deformity with a global incidence rate of 3% [2]. AIS involves excessive coronal curvature, incorrect axial spine rotation, and sagittal deformity, impacting approximately 5% of the world's population [3]. Its etiology remains largely unknown and does not appear to be related to neuromuscular, syndromic, or congenital conditions [4].

Historically, the management of moderate and mild AIS primarily included observation and bracing, with surgical intervention reserved for patients exhibiting severe curvature [5]. Various interventions have been established to correct scoliosis deformities, halt disease progression, and enhance the quality of life for AIS patients [6]. Treatment options range from no intervention and non-surgical management, such as bracing and physical therapy, to surgical procedures, including posterior spinal fusion (PSF) and vertebral body tethering (VBT) [7, 8]. The choice of treatment largely depends on the severity of the spinal curve and the consideration of the patient or family.

PSF remains the traditional method for addressing scoliosis by fusing the spine through pedicle screw instrumentation, particularly for patients with advanced Cobb angles who exhibit radiographic worsening and clinical imbalance following conservative treatment [9, 10]. However, concerns have emerged regarding the implications of spinal fusion, including growth restriction, decreased spinal flexibility, loss of motion, stiffening in the operated area, and alterations in biomechanics [11, 12]. In contrast, VBT has been introduced as a novel surgical technique focused on growth modulation, effectively circumventing some of the functional complications associated with PSF [13]. VBT is a non-fusion procedure that employs an anterior approach, utilizing vertebral body screws and a cable to compress the convex side of the curve, thereby correcting idiopathic scoliosis while minimizing potential functional complications [14, 15]. This innovative method allows adolescents to take advantage of their remaining spinal growth to correct scoliotic deformities [16], thereby preserving functional motion and achieving a more normal spinal contour.

Despite its advantages, VBT presents challenges, including risks of overcorrection, procedural failure, and construct breakage, which may necessitate revision surgeries or even conversion to PSF [17]. Moreover, the literature on PSF and VBT remains fragmented, highlighting the need for a comprehensive meta-analysis of the existing research.

This meta-analysis aims to compare PSF and VBT in the management of AIS, focusing on both radiographic and patient-reported outcomes. The primary outcomes include the degree of curve correction, revision rates, and complication rates. Secondary outcomes include functional outcomes and patient-reported quality of life (e.g., Scoliosis Research Society-22 (SRS-22) scores). This work seeks to clarify the relative strengths and limitations of each approach to support evidence-based clinical decision-making.

## Review

Study design

The systematic review and meta-analysis were conducted to compare PSF and VBT in the management of AIS. The study adhered to the guidelines set forth by the Preferred Reporting Items for Systematic Reviews and Meta-Analyses (PRISMA) 2020 and the Quality of Reporting of Meta-analyses (QUORUM) to ensure clarity and reproducibility. It was registered on the International Prospective Register of Systematic Reviews (PROSPERO) database (PROSPERO ID: CRD420251006260). This article was previously posted to the Research Square preprint server on 16 April 2025.

Search strategy

A comprehensive literature search was conducted across four major electronic databases: PubMed (Medical Literature Analysis and Retrieval System Online (MEDLINE)), Scopus, Cochrane Library, and Google Scholar. The search covered publications from database inception through 2024. Boolean operators (AND, OR, NOT) were used to combine search terms, including 'posterior spinal fusion,' 'vertebral body tethering,' 'adolescent idiopathic scoliosis,' 'curve correction,' 'complication rates,' and 'functional outcomes.' Search strategies were tailored for each database to include Medical Subject Headings (MeSH) and free-text keywords. A manual search of reference lists from included studies was also conducted to identify any additional relevant studies not captured during the database search. Inclusion and exclusion criteria are shown in Table 1.

**Table 1:** Inclusion and exclusion criteria PSF: posterior spinal fusion; VBT: vertebral body tethering; RCTs: randomized controlled trials

Inclusion criteria	Exclusion Criteria
Studies comparing PSF and VBT in adolescents with idiopathic scoliosis, published in English in peer-reviewed journals, reporting on curve correction, complication rates, quality of life, or functional outcomes; follow-up period of ≥2 years; Study designs: RCTs, cohort, prospective, or retrospective studies	Studies not comparing PSF and VBT; studies on non-adolescent populations or other scoliosis types; Follow-up less than two years; conference abstracts, grey literature, or non-peer-reviewed articles; non-English language publications

Study selection process

**Figure 1:**
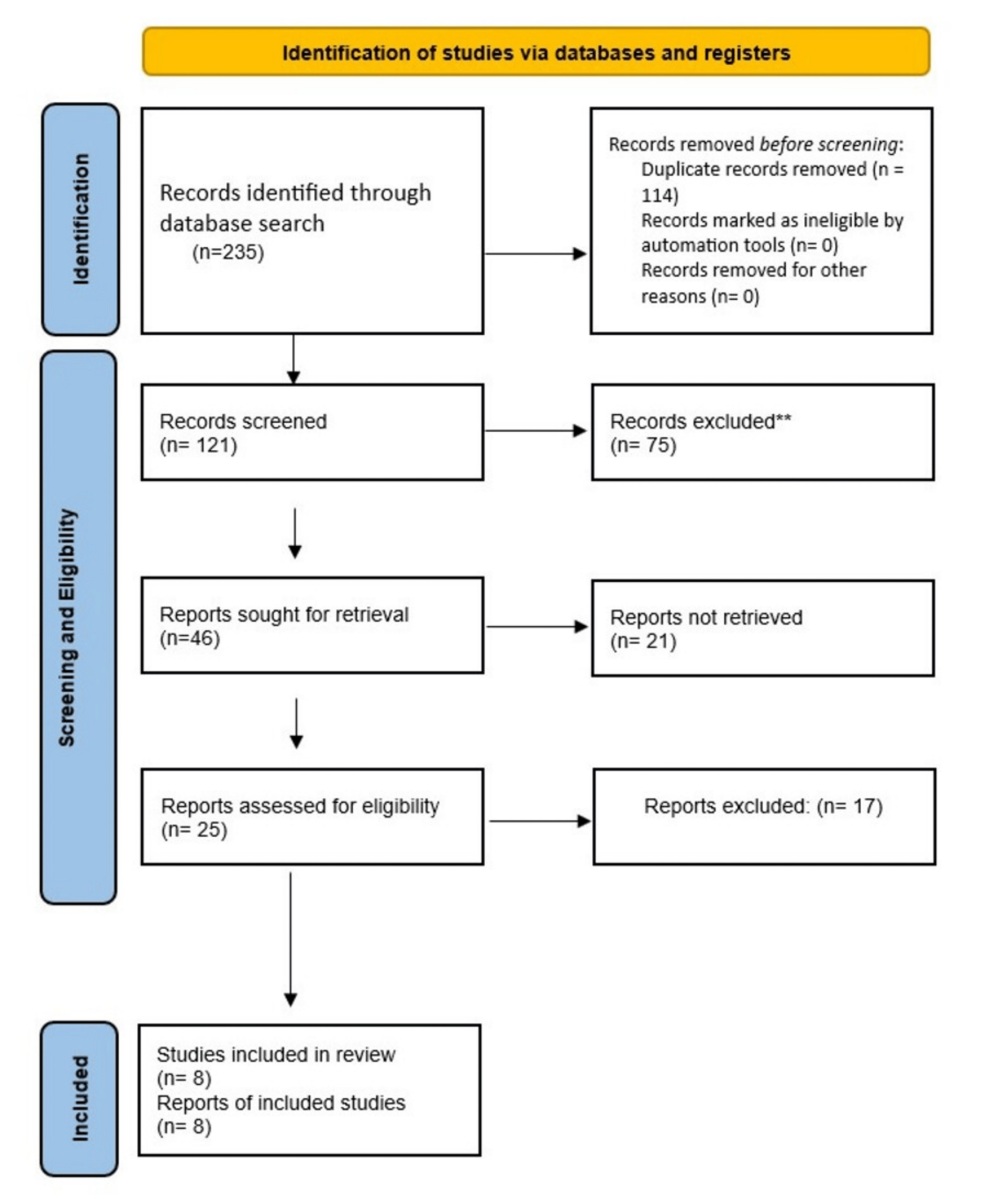
A PRISMA flowchart outlining the study selection process PRISMA: Preferred Reporting Items for Systematic Reviews and Meta-Analyses

The selection process followed PRISMA guidelines. After duplicates were removed, titles and abstracts were screened for relevance. Full-text articles were then reviewed against the inclusion and exclusion criteria. Three independent reviewers performed the selection process, and disagreements were resolved by consensus or with input from a fourth reviewer. Reference management was conducted using Mendeley (Elsevier, Amsterdam, Netherlands). The PRISMA flow diagram in Figure 1 illustrates the selection process, including the number of studies identified, screened, and included.

Data analysis

Quantitative data were analyzed. For continuous outcomes, standardized mean differences (SMDs) were calculated, while dichotomous outcomes were assessed using odds ratios (ORs), both with 95% confidence intervals (CIs). A random-effects model was applied due to anticipated heterogeneity. Forest plots were generated to illustrate individual and pooled effect sizes. Heterogeneity was assessed using the I² statistic, with thresholds of 25%, 50%, and 75% representing low, moderate, and high heterogeneity, respectively.

Publication bias was evaluated using funnel plots and Egger’s test. Where bias was detected, the trim-and-fill method was employed to adjust for its impact. Subgroup analyses were performed to explore heterogeneity sources, focusing on patient age, curve severity, and surgical approach. Sensitivity analyses excluded studies with a high risk of bias to assess the robustness of findings.

Data extraction and synthesis

Data were independently extracted by reviewers using a standardized, pretested extraction form. The extracted data included study characteristics, participant demographics, interventions, outcomes, and results relevant to curve correction, complication rates, quality of life, and reoperation rates. Discrepancies were resolved through discussion or third-party adjudication. A comprehensive analysis was then conducted and visually presented using forest plots, with subgroup and sensitivity analyses to ensure robust conclusions.

Results

In this meta-analysis, PSF and VBT for AIS were examined for curve rectification, intraoperative and postoperative complications, functional results, and patient-centered metrics. Two approaches for treating AIS-related spinal abnormalities were tested in this study. A total of eight studies were selected following the PRISMA flowchart (Table 2).

**Table 2:** Data extraction summary from the included papers AVBT: anterior vertebral body tethering; PSF: posterior spinal fusion; QoL: quality of life; VBT: vertebral body tethering; HRQoL: health-related quality of life; AIS: adolescent idiopathic scoliosis; T curve: thoracic curve; TL: thoracolumbar

Citation	Study Aim	Method	Key Findings	Relevance
Newton et al. (2020) [18]	Compare AVBT vs. PSF in thoracic AIS patients over two to five years	Retrospective; 49 patients; minimum two-year follow-up	PSF had better correction (16° vs 33°); nine revisions in AVBT, none in PSF	PSF is more effective in maintaining correction, fewer revisions.
Pehlivanoglu et al. (2021) [19]	Compare clinical/functional outcomes and QoL in AIS after VBT vs. PSF	Retrospective; 43 patients; minimum three-year follow-up	VBT was superior in motion, strength, satisfaction, and QoL scores.	VBT shows functional and quality-of-life advantages
Qiu et al. (2021) [20]	Compare HRQoL outcomes between AVBT and PSF	Comparative: 82 patients (62 PSF, 20 AVBT)	Similar HRQoL; AVBT had more thoracic flexibility (59% vs. 43%)	VBT preserves flexibility; outcomes are equivalent in HRQoL.
Mathew et al. (2022) [21]	Compare two-year outcomes of VBT vs. PSF in skeletally immature AIS	Prospective matched case-control; 26 VBT & 26 PSF	PSF had better correction (66% vs 46%); VBT showed continued growth	PSF corrects more; VBT preserves growth
Newton et al. (2022) [22]	Large multicenter comparison of AVBT vs. PSF using propensity matching	Retrospective; 237 matched pairs	PSF had better correction and fewer revisions (1.3% vs. 16%)	PSF provides better long-term stability and fewer re-operations
Pahys et al. (2022) [23]	Compare trunk motion between AVBT and PSF using 3D kinematics	Retrospective; 112 patients; two-year follow-up	AVBT had less motion loss; PSF showed greater trunk stiffness	AVBT preserves motion better than PSF
Siu et al. (2023) [24]	Compare perioperative, pain, and clinical outcomes of AVBT vs. PSF	Retrospective cohort; two-year follow-up	PSF had greater correction; AVBT had less blood loss and operation time	PSF offers better correction; AVBT is less invasive
Lonner et al. (2024) [25]	Evaluate outcomes in double-curve AIS with VBT vs. PSF	Comparative: 29 patients	PSF better in T curves; similar outcomes in TL curves; VBT had more complications	PSF is preferred for T curves; TL outcomes similar

This study compared the radiographic outcomes, complication rates, and patient satisfaction associated with PSF and VBT. The analysis incorporated descriptive statistics, distributional comparisons, and statistical tests to evaluate the efficacy and safety of each procedure.

The key radiographic and clinical outcomes analyzed included the preoperative curve magnitude, the degree of postoperative curve correction, revision rates, and complication rates. As shown in Table 3, PSF resulted in a higher mean curve correction (79.0) compared to VBT (51.0), suggesting a more effective deformity correction overall. Although the mean preoperative curve was slightly higher for PSF patients (54.0) than for VBT patients (51.5), this difference did not appear to impact correction outcomes negatively. Additionally, PSF was associated with lower mean revision and complication rates (17.0 for both) relative to VBT, where both rates were higher at 27.8. These differences suggest that PSF may yield not only superior radiographic outcomes but also fewer post-surgical issues.

**Table 3:** Descriptive statistics for primary outcomes PSF: posterior spinal fusion; VBT: vertebral body tethering

Outcome	PSF	VBT
Preoperative curve (mean)	54	51.5
Preoperative curve (SD)	0	1.57
Correction (Mean)	79	51
Correction (SD)	13.14	18.43
Revision rate (mean)	17	27.8
Revision rate (SD)	14.23	18.43
Complication rate (mean)	17	27.8
Complication rate (SD)	15.54	15.54

**Figure 2:**
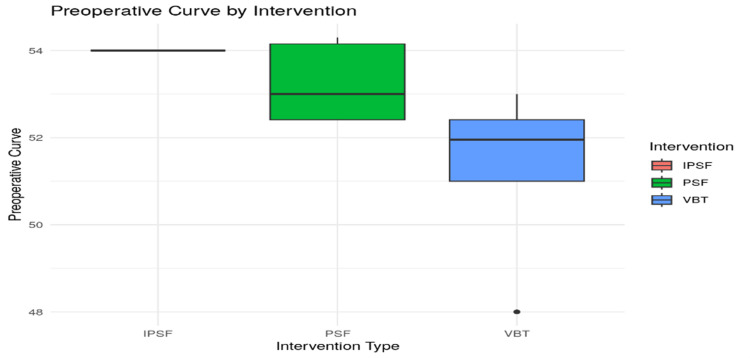
Distribution of preoperative curve for PSF and VBT IPSF: instrumented posterior spinal fusion; PSF: posterior spinal fusion; VBT: vertebral body tethering

**Figure 3:**
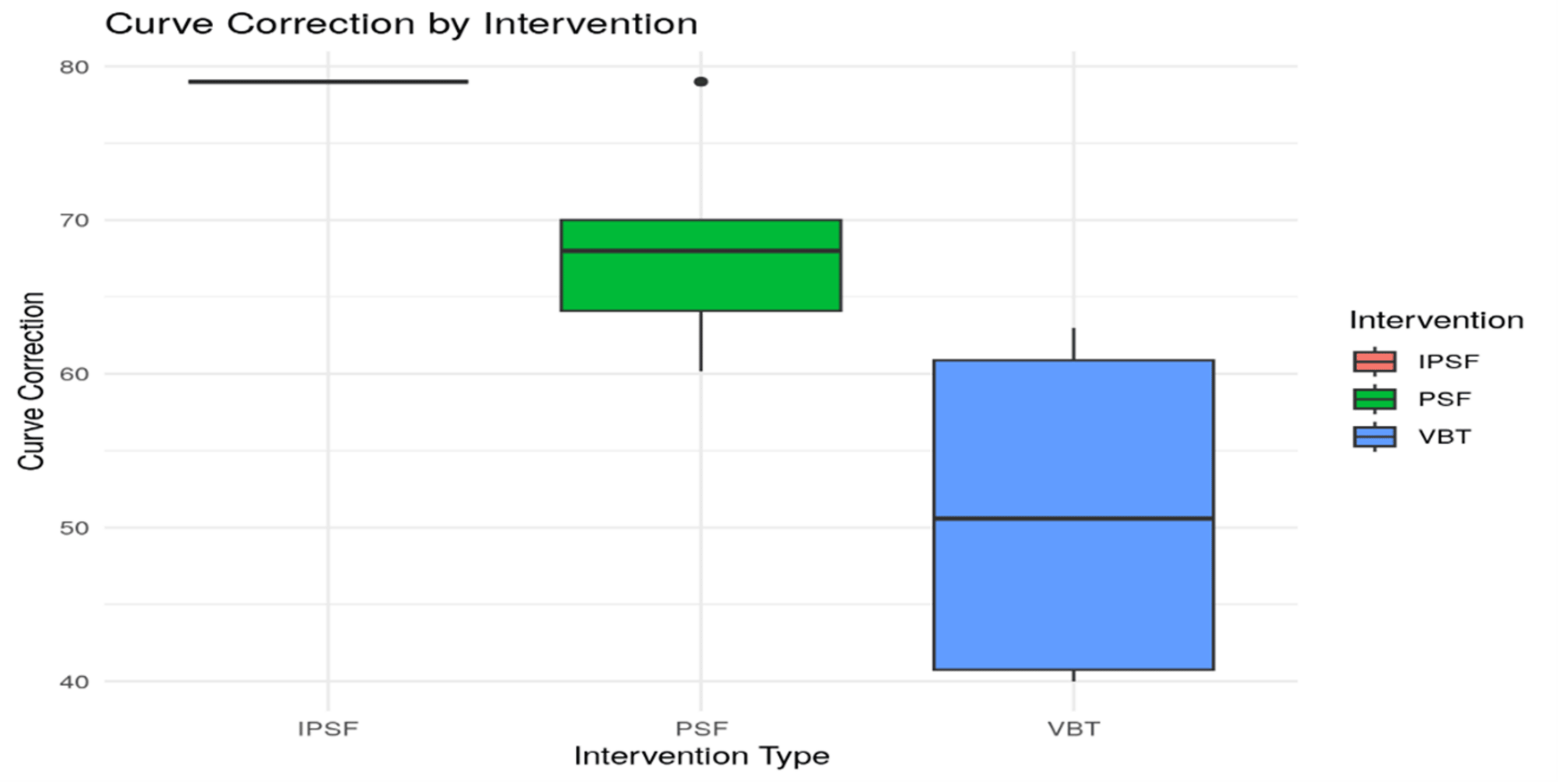
Distribution of curve correction for PSF and VBT IPSF: instrumented posterior spinal fusion; PSF: posterior spinal fusion; VBT: vertebral body tethering

Visual comparisons of the distribution of preoperative curves and correction magnitudes are provided in Figures 2, 3. The PSF group showed a tighter interquartile range in their preoperative curve distribution, indicating less variability in baseline deformities (Figure 2). In terms of correction, PSF again exhibited higher median values and less dispersion, confirming its clinical superiority in achieving consistent spinal alignment (Figure 3). The VBT group, in contrast, demonstrated broader variability and lower correction values.

**Figure 4:**
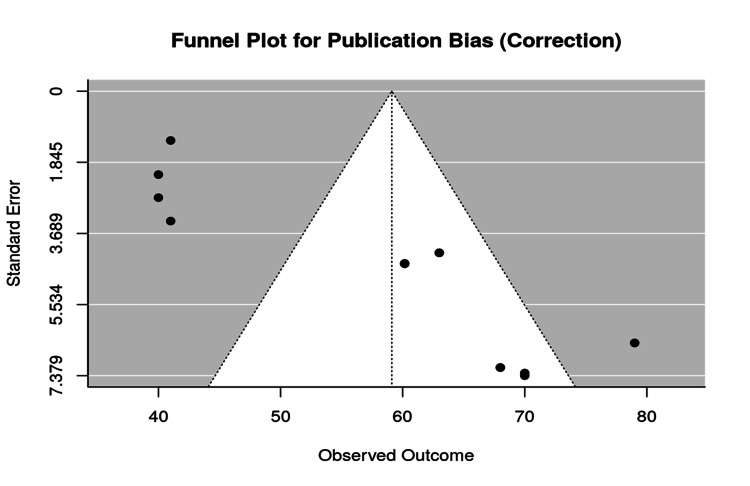
Funnel plot for correction (assessment of publication bias)

**Figure 5:**
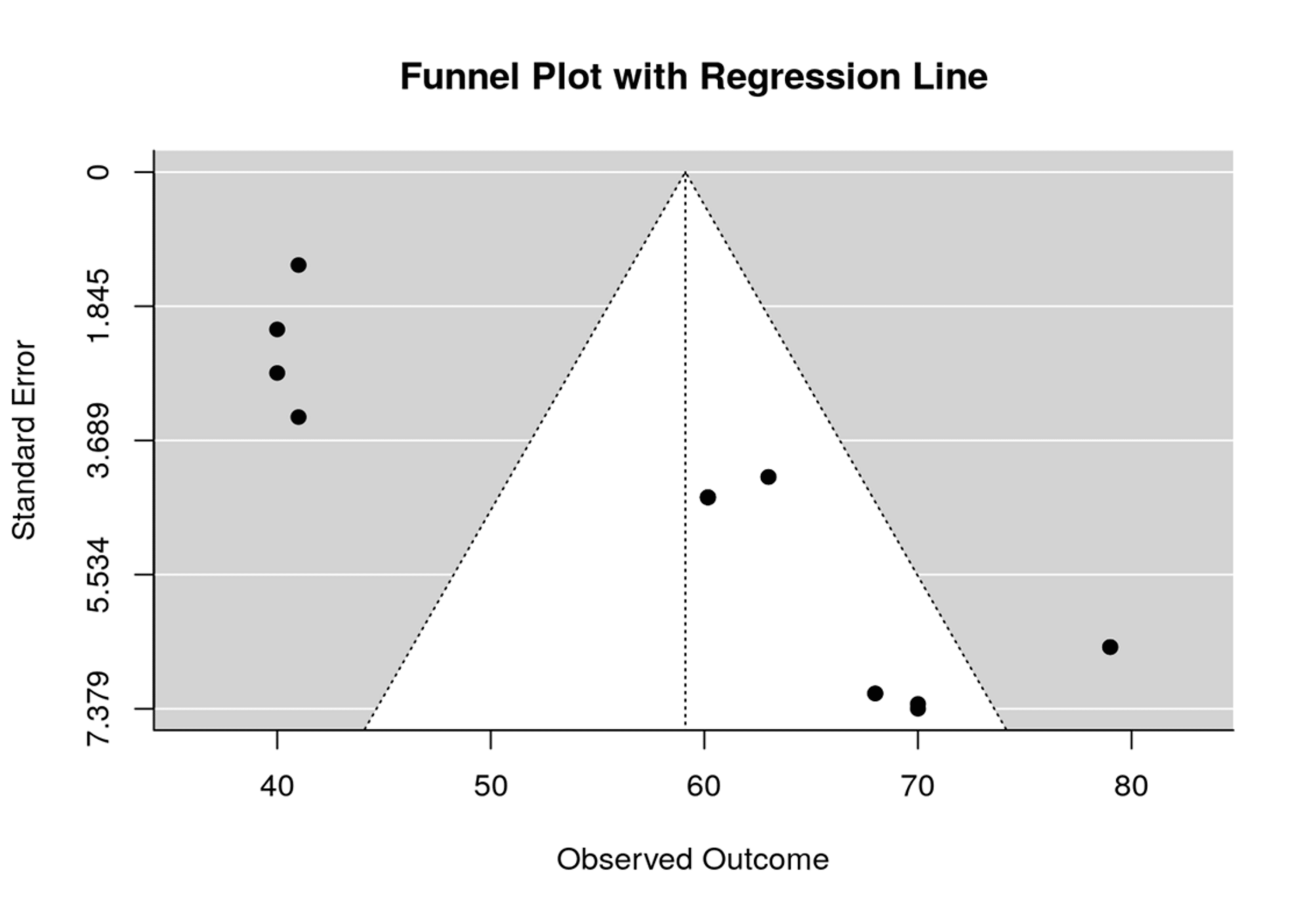
Funnel plot with regression line (bias symmetry analysis)

To evaluate potential publication bias, a funnel plot was generated based on the correction variable (Figure 4). While some asymmetry was noted, the overall shape remained sufficiently balanced around the central effect size. The addition of a regression line in Figure 5 revealed a weak slope, suggesting minimal bias.

**Figure 6:**
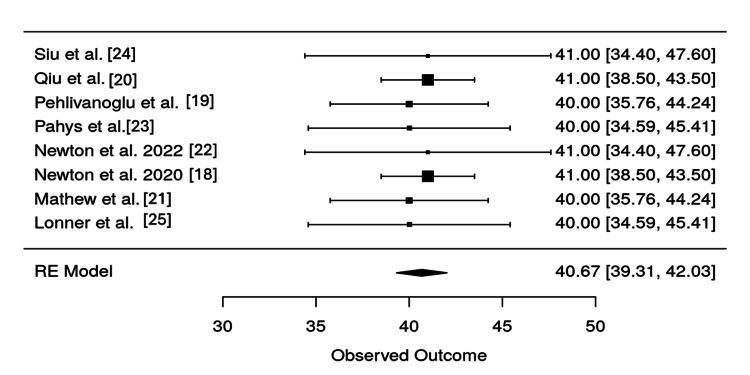
Forest plot of correction outcomes across studies

Heterogeneity testing, as summarized in Table 4, confirmed consistency across studies. The I² statistic was 0%, and the p-value for the Q-test was 0.9996, indicating no significant heterogeneity. The overall estimate for correction was 40.67, with a 95% confidence interval of (39.31, 42.03), and the result was highly statistically significant (p < 0.0001). These findings are visually supported by the forest plot in Figure 6, where individual and pooled study outcomes are aligned closely, indicating limited variation.

**Table 4:** Heterogeneity test results

Statistic	Value
I²	0%
Q-value	0.462
p-value for heterogeneity	0.9996
Overall estimate (correction)	40.67
95% CI	(39.31, 42.03)
p-value for estimate	<0.0001

Tests of normality and homogeneity of variances, shown in Tables 5, 6, revealed that all four key variables, such as preoperative curve, correction, revision rate, and complication rate, were non-normally distributed according to the Shapiro-Wilk test (p < 0.05). Levene’s test indicated homogeneous variances for all variables except correction, which exhibited variance heterogeneity. These results justified the use of non-parametric tests such as the Mann-Whitney U test, and Welch’s t-test was employed for the correction outcome due to variance inconsistency.

**Table 5:** Shapiro-Wilk normality test

Variable	W Statistic	p-value	Interpretation
Preoperative curve	0.8612	0.02	Not normally distributed
Correction	0.8759	0.0336	Not normally distributed
Revision rate	0.8469	0.0123	Not normally distributed
Complication rate	0.7467	0.0006	Not normally distributed

**Table 6:** Levene’s test for homogeneity of variance

Variable	F Value	p-value	Variance homogeneity
Preoperative curve	0.7106	0.5095	Homogeneous
Correction	9.9874	0.0024	Heterogeneous
Revision rate	1.9074	0.1878	Homogeneous
Complication rate	0.2373	0.7921	Homogeneous

**Figure 7:**
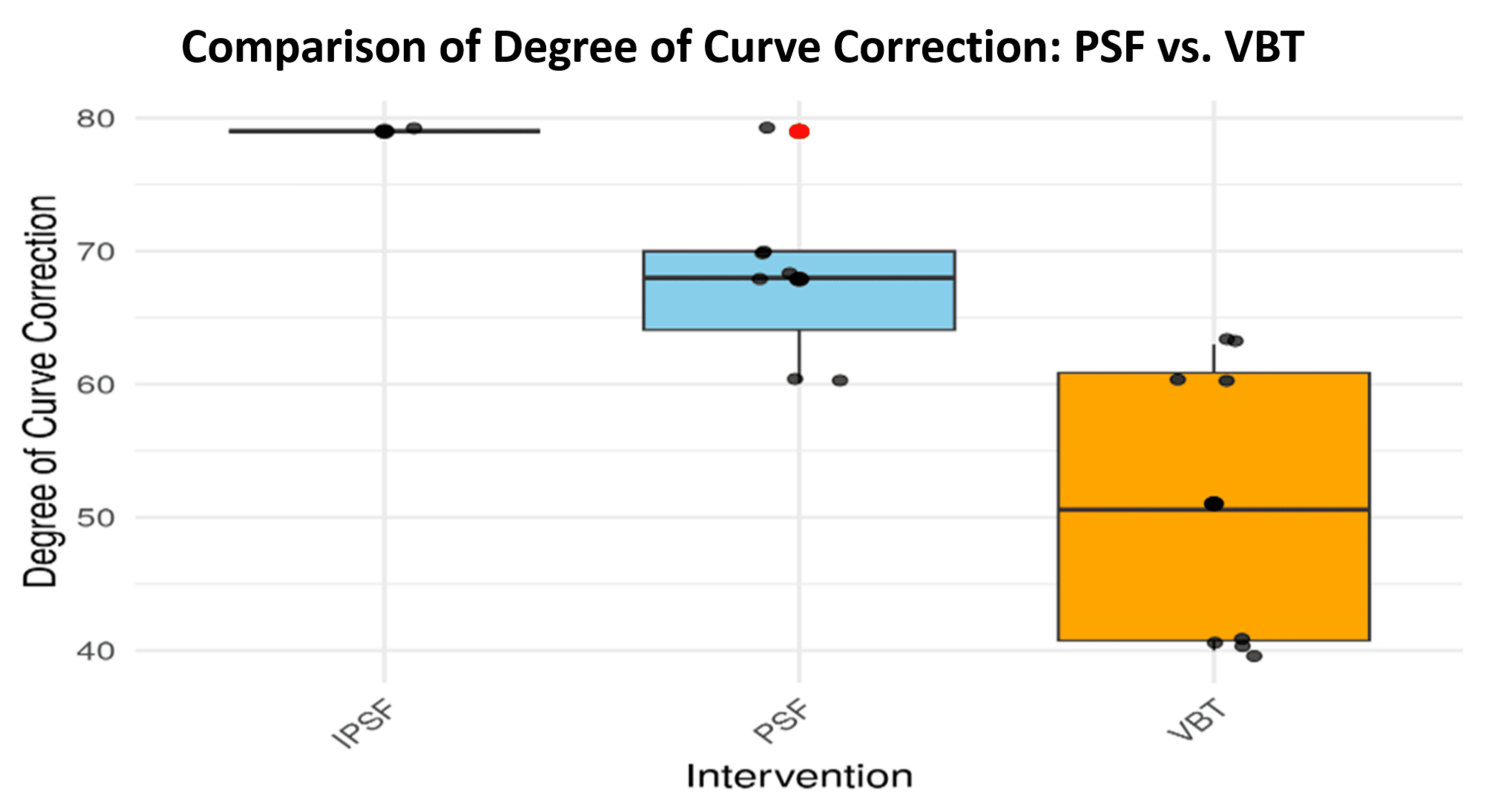
Boxplot comparison of curve correction between PSF and VBT IPSF: instrumented posterior spinal fusion; PSF: posterior spinal fusion; VBT: vertebral body tethering

Curve correction was further analyzed statistically using the Wilcoxon rank sum test, and the results are presented in Table 7. The test revealed a statistically significant difference between PSF and VBT correction values (W = 50, p = 0.0117). The distribution is visually depicted in Figure 7, where PSF shows greater correction values, more variability, and a few notable outliers, while VBT exhibits a lower and more uniform range.

**Table 7:** Statistical comparison for curve correction between PSF and VBT PSF: posterior spinal fusion; VBT: vertebral body tethering

Test	Statistic	p-value	Interpretation
Shapiro-Wilk test (PSF)	W = 0.89373	0.2947	Normal distribution assumption holds
Shapiro-Wilk test (VBT)	W = 0.73149	0.005088	Normal distribution assumption does not hold
Levene's test	p-value = 0.00224	0.00224	Variances are not homogeneous
Wilcoxon rank sum test	W = 50	0.01166	Significant difference in curve correction between PSF and VBT

**Figure 8:**
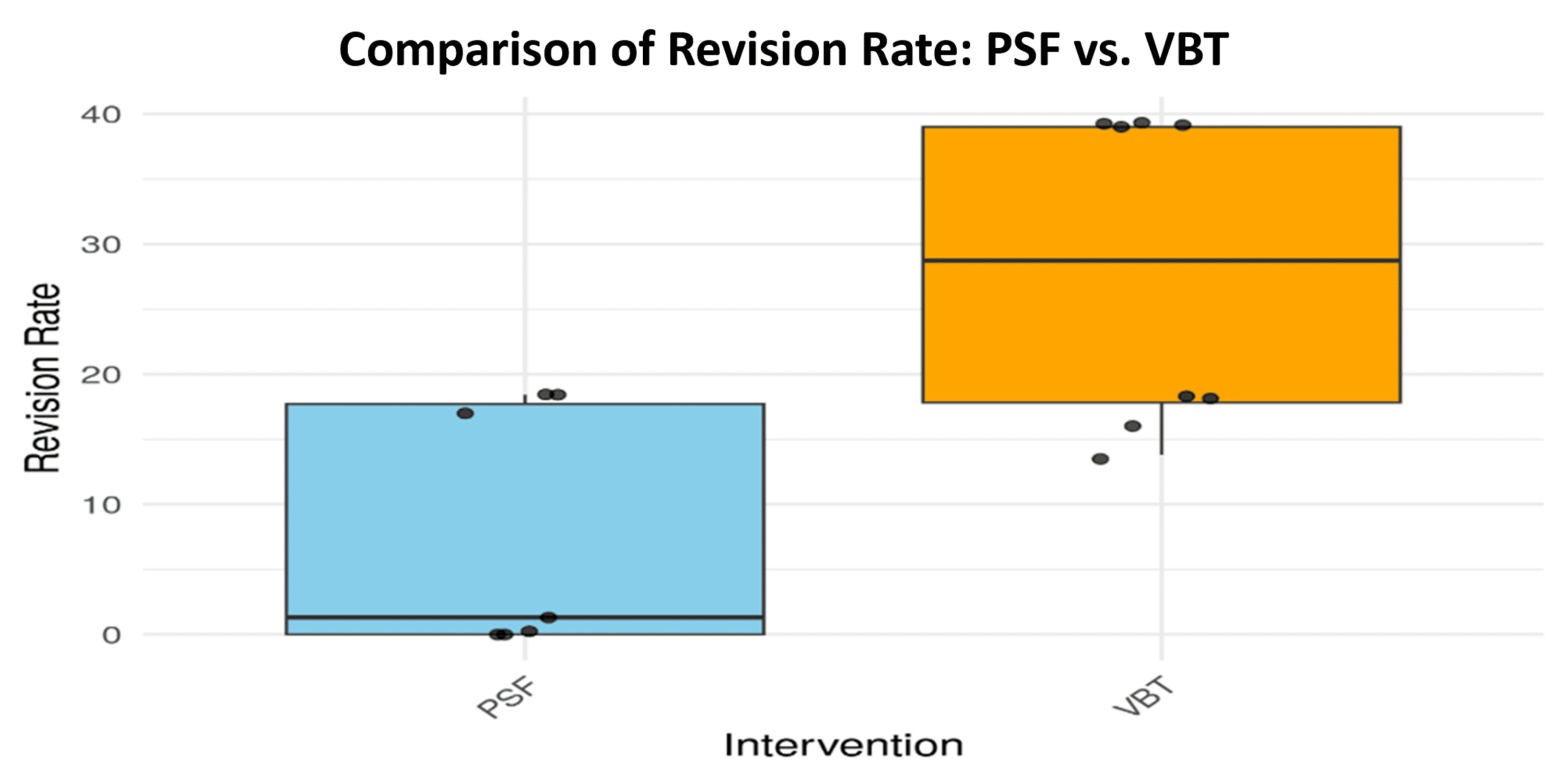
Revision rates comparison between PSF and VBT PSF: posterior spinal fusion; VBT: vertebral body tethering

**Figure 9:**
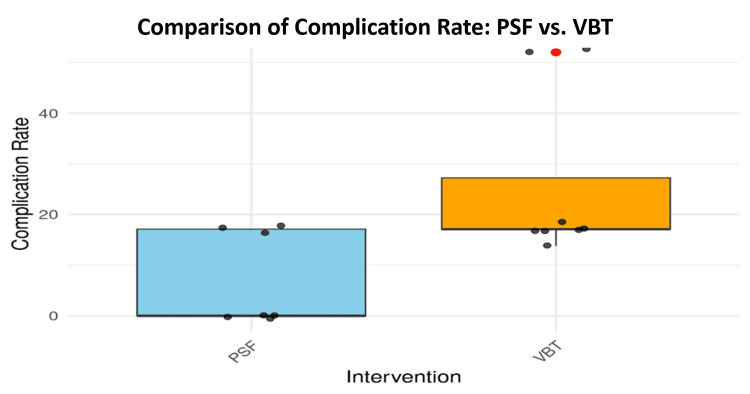
Complication rates comparison between PSF and VBT PSF: posterior spinal fusion; VBT: vertebral body tethering

Postoperative revision and complication rates were then compared. Significant differences were found using the Wilcoxon Rank Sum Test, with PSF demonstrating lower revision rates (W = 8, p = 0.0211) and fewer complications (W = 9, p = 0.0225). These findings are clearly visualized in Figures 8, 9, respectively.

**Figure 10:**
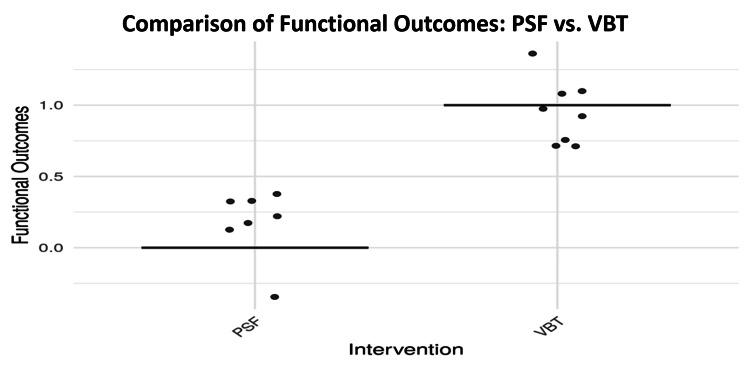
Comparison of functional outcomes between PSF and VBT PSF: posterior spinal fusion; VBT: vertebral body tethering

**Figure 11:**
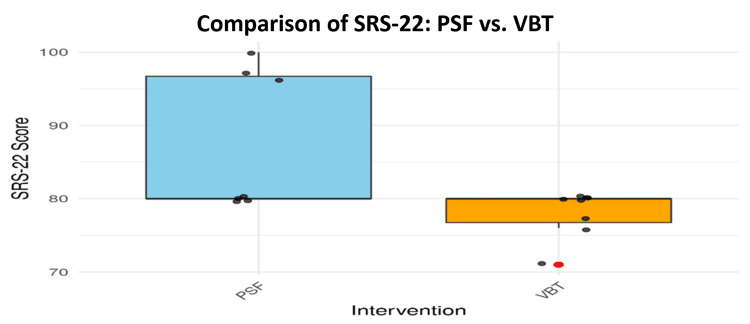
Comparison of SRS-22 patient satisfaction scores between PSF and VBT SRS-22: Scoliosis Research Society-22; PSF: posterior spinal fusion; VBT: vertebral body tethering

The study also examined patient-reported functional outcomes and satisfaction using the Scoliosis Research Society-22 (SRS-22) score questionnaire. As detailed in Table 8, the Mann-Whitney U test showed significant differences in both functional outcomes and SRS-22 scores (p = 0.000238), and the Welch’s t-test also confirmed a significant difference in satisfaction levels (p = 0.0188). These findings were visualized in Figures 10, 11. The functional outcomes plot (Figure 10) suggests higher functional recovery among VBT patients, albeit with greater variability and outliers. In contrast, Figure 11 shows more consistent and higher satisfaction scores among PSF patients, reinforcing its more uniform postoperative quality of life benefits.

**Table 8:** Statistical Tests for Functional Outcomes and SRS-22 SRS-22: Scoliosis Research Society-22; PSF: posterior spinal fusion; VBT: vertebral body tethering

Test	Statistic	p-value	Interpretation
Mann-Whitney U Test for functional outcomes	W = 0	0.000238	Significant difference in functional outcomes between PSF and VBT
Mann-Whitney U Test for SRS-22	W = 0	0.000238	Significant difference in SRS-22 between PSF and VBT
Welch's t-test for SRS-22	t = 2.6823	0.01882	Significant difference in means of SRS-22 between PSF and VBT

PSF demonstrated superior curve correction, fewer revisions and complications, and more consistent patient satisfaction. While VBT showed potential advantages in postoperative mobility, it was associated with increased variability in outcomes and a higher rate of adverse events. These findings emphasize the importance of individualized treatment selection in AIS and highlight the clinical reliability of PSF across multiple domains of outcome assessment.

Discussion

This comparative analysis between PSF and VBT aimed to assess the effectiveness and outcomes of each technique in treating AIS.

A central focus of the analysis was the degree of curve correction achieved through each surgical intervention. Several statistical tests were applied to evaluate this outcome, with the Wilcoxon rank sum test producing a statistically significant result (p = 0.01166), indicating that PSF was more effective than VBT in correcting the major thoracic curve. This finding aligns with the work of Yucekul and Ergene (2021), who reported that PSF provides a more immediate and pronounced rectification of scoliosis curvature, particularly in the thoracic spine [26].

Interestingly, while PSF demonstrated greater correction efficacy, the VBT group showed reduced variability across patients. This consistency in outcomes was supported by the narrower interquartile range observed in the VBT data (Figure 7), a point also emphasized by Oeding et al. (2023), who noted the relative uniformity of VBT outcomes across patient populations [27]. VBT patients were observed to return to physical activity, including sports, more rapidly; however, their ability to correct both thoracic and lumbar spinal curves remained less effective than PSF. The design of VBT allows for ongoing spinal growth, an advantage in skeletally immature patients, but PSF demonstrated a more substantial mechanical correction of spinal deformity. Outliers in the PSF group, represented by red data points in Figure 7, suggest that some individuals benefited from dramatic improvements in curvature, highlighting its potential for strong corrective results in select cases.

Nevertheless, such aggressive corrections can also bring about challenges. As Shin et al. (2021) documented, the significant curve rectification delivered by PSF may, in certain cases, lead to excessive spinal rigidity [28]. This complication can result in limitations to mobility and, in some younger patients, lead to long-term functional impairment. These risks are especially pronounced in patients under 18 years of age or those presenting with more severe curvatures.

In terms of intraoperative and postoperative complications, the study evaluated both complication and revision rates as distinct clinical outcomes. As shown in Figure 8, PSF patients experienced significantly fewer revision surgeries compared to VBT patients, a difference substantiated by a p-value of 0.02107. Shin et al. (2021) also highlighted this trend, noting that while VBT preserves spinal growth, it carries a higher risk of complications, particularly tether failures that may necessitate subsequent operations. Supporting this, Hoernschemeyer et al. (2020) found that approximately 50% of patients undergoing VBT required tether repair, with the procedure resulting in a revision rate of 21% [28, 29].

Data presented in Figure 9 illustrate that PSF patients experienced fewer issues such as hardware failure, surgical site infections, and overall postoperative complications. These findings are corroborated by Eaker et al. (2024), who reported that VBT procedures, while beneficial for growth preservation, often come with increased instability due to tether-related failures [30]. Such complications are particularly common in younger, skeletally immature patients who are often the primary candidates for VBT, posing a challenge in long-term postoperative management. In contrast, PSF was found to offer greater long-term stability with fewer adverse events, making it a safer option for spinal fusion in AIS cases requiring sustained correction.

The analysis of functional outcomes and patient satisfaction revealed significant differences between the surgical methods. Both the Welch’s t-test and the Mann-Whitney U test confirmed that patients who underwent VBT reported better postoperative functional outcomes than those treated with PSF, with a p-value of 0.000238 supporting this statistically significant distinction. Patient-reported outcome measures, specifically SRS-22 scores, suggested that VBT recipients experienced enhanced postoperative mobility and greater satisfaction in the months following surgery.

Despite the superior functional performance observed in the VBT group, the PSF group demonstrated more consistent improvements in overall quality of life and patient satisfaction. This is visualized in Figure 11, where PSF patients showed more stable median SRS-22 scores with fewer outliers, indicating uniform satisfaction across the group. Yucekul and Ergene (2021) observed that, while VBT did not deliver the immediate curve correction seen with PSF, it did lead to gradual improvements in functionality and quality of life [26]. Patients in the PSF group, however, followed more predictable recovery trajectories and benefited from improved consistency in clinical outcomes. This predictability is one of the key factors contributing to the higher satisfaction levels reported.

Furthermore, Figure 10 presents functional recovery results, where PSF patients exhibited superior SRS-22 median scores, denoting better overall quality of life. This is in line with Oeding et al. (2023), who reported that PSF patients often experience expedited recovery that enables participation in sports and other physical activities, thereby enhancing postoperative contentment [27]. While VBT patients sometimes reported better flexibility, this advantage came with increased variability in individual experiences.

Regarding postoperative mobility and reoperation rates, the study identified that PSF led to fewer reoperations than VBT. This is again highlighted in Figure 8, where the PSF group required significantly fewer surgical revisions. The findings are consistent with Shin et al. (2021), who described elevated reoperation rates among VBT patients, often triggered by complications such as tether breakage or overcorrection [28]. These issues raise concerns about the long-term durability of VBT and its appropriateness for all patients, especially those who may need extended spinal stabilization.

On the other hand, studies by Oeding et al. (2023) showed that VBT patients occasionally displayed greater postoperative mobility during follow-up assessments, indicating some functional benefits [27]. However, the VBT group’s recovery patterns were noted to be less predictable and more individualized. In contrast, PSF patients showed consistent recovery progress, though often accompanied by reduced spinal flexibility and increased stiffness over time: a pattern also described by Hoernschemeyer et al. (2020) [29]. This rigidity is a trade-off for the durable correction offered by PSF and is important to consider during surgical planning.

When considering the strengths and limitations of the included studies, it is evident that the use of multiple high-quality studies contributed to a higher level of statistical power and robustness in the results. The inclusion of multiple datasets allowed for more reliable conclusions than could be drawn from any single study alone.

However, certain limitations must be acknowledged. Not all of the studies provided complete data for inclusion, particularly regarding intraoperative and postoperative complications. For instance, studies by Pehlivanoglu et al. (2021), Qiu et al. (2021), and Pahys et al. (2022) did not report sufficient information regarding adverse events associated with either VBT or PSF, limiting their value for certain outcome comparisons [19, 20, 23].

Additionally, although efforts were made to assess potential publication bias, the funnel plot and regression line (previously shown in Figures 4, 5) suggested some asymmetry in the data. While this may point to the existence of mild bias, such as selective reporting of studies with favorable outcomes, the distribution of data points was reasonably fair across the range. The slight asymmetry does not appear to introduce significant distortion into the study's overall findings.

## Conclusions

This meta-analysis highlights the importance of individualized surgical planning for AIS. PSF remains a strong and reliable approach for achieving substantial spinal correction with long-term stability. However, its use may reduce spinal flexibility, potentially affecting functional outcomes over time. VBT provides an alternative that maintains more natural motion and supports spinal growth during adolescence. This approach can be beneficial for younger patients prioritizing mobility, but carries a greater likelihood of requiring additional procedures. Selecting the most appropriate intervention should therefore consider factors such as patient age, skeletal maturity, curve characteristics, and desired long-term functional results.
